# A “willingness to be orchestrated”: Why are UK diplomats working with tobacco companies?

**DOI:** 10.3389/fpubh.2023.977713

**Published:** 2023-03-17

**Authors:** Raouf Alebshehy, Karin Silver, Phil Chamberlain

**Affiliations:** Department for Health, University of Bath, Bath, United Kingdom

**Keywords:** Framework Convention on Tobacco Control (FCTC), international law, tobacco industry, Article 5.3, diplomat, lobbying, foreign affairs, LMICs

## Abstract

**Background:**

The tobacco epidemic is global and addressing it requires global collaboration. International and national policies have been adopted to promote collaboration for tobacco control, including an obligation on diplomatic missions to protect public health from the vested interests of the tobacco industry. However, incidents of diplomats engaging with the tobacco industry are still occurring despite these regulations. This paper presents a case study of a British ambassador actions, and it points to some of the challenges researchers face in monitoring such incidents.

**Methods:**

The incident studied in this paper was first identified through regular media monitoring conducted by the Tobacco Control Research Group at the University of Bath. The incident was further investigated by using the tools made available by the United Kingdom (UK) Freedom of Information Act, including submitting a request, asking for internal review, and submitting a complaint to the Information Commissioner's Office.

**Results:**

We identified clear evidence of the UK ambassador to Yemen opening a cigarette factory, part owned by British American Tobacco (BAT), in Jordan. Our investigation revealed a lack of documentation of this and similar incidents of interaction between diplomats and the tobacco industry. We raise concerns about the actions of diplomats which contravene both national and international policies.

**Discussion:**

Monitoring and reporting such activities produces several challenges. Diplomats' interactions with the tobacco industry represent a major concern for public health as such interactions seem to be systematically repeated. This paper calls for action to better implement national and international policies to protect the public health including in low- and middle-income countries (LMICs).

## Introduction

There are multiple documented incidents of diplomatic representatives acting contrary to national and international law and apparently lobbying for the tobacco industry, particularly in Low- and Middle-Income Countries (LMICs), and the evidence suggests that diplomats, including ambassadors, have been utilized in many countries to serve the commercial interests of tobacco companies (1–4). While the activity has been documented worldwide, this strategy for interference is much more concentrated, originating largely in the big transnational tobacco companies. It is no coincidence that British American Tobacco (BAT), Philip Morris International and Japan Tobacco all have a presence in Geneva, the home of the World Health Organization and other key international organizations including the World Trade Organization (WHO) and the International Labor Organization, all arenas where tobacco companies aim to exert influence (5).

This paper gives a summary of incidents where diplomats have contributed towards the promotion of tobacco companies, often in contravention of obligations under the WHO Framework Convention on Tobacco Control (WHO FCTC) (6). We detail a case of a United Kingdom (UK) ambassador engaging with the tobacco industry in Jordan and Yemen. We suggest such actions privilege business interests over those of public health. The exposure of such activities, and the continuous monitoring of the tobacco industry, is a requirement of the WHO FCTC. We make recommendations for research and policy change, including more rigorous documentation of diplomats' engagement with the industry. This would inform policy makers of the policies necessary to protect countries, especially LMICs, from such incidents.

## Background

Tobacco control regulations vary from one country to another. In national level tobacco control acts, the main aim is to protect public health within the jurisdiction of the country, without much focus on other populations. Therefore, there is usually an absence of policies regulating diplomats' actions abroad. However, the tobacco epidemic is a global one and therefore cross-border measures have been adopted by governments to tackle it. Additionally, there are international regulations which, while not specific to tobacco, are applicable to the tobacco control context. Relevant national and international regulations are summarized in **Box 1**.

Box 1 **Regulations related to diplomats and engagement with tobacco industry.**

International law

Under international law, tobacco control measures are covered by two treaties: the World Health Organization Framework Convention on Tobacco Control (WHO FCTC), entered into force in 2005; and the Protocol to Eliminate Illicit Trade in Tobacco Products, entered into force in 2018. The WHO FCTC is one of the most rapidly and widely embraced treaties in United Nations history with 182 Parties (6).

Article 2 of the WHO FCTC states that it respects its Parties rights and obligations under other international agreements if they are compatible with the Parties' obligations under the WHO FCTC. In Article 4, the WHO FCTC recognizes the importance of international cooperation in establishing and implementing effective tobacco control measures. It highlights the need for comprehensive multisectoral measures to tackle the epidemic at international levels. This points to the need for a leading role from diplomatic missions to promote such collaboration among different countries.

In this context, it is important to flag the Vienna Convention on Diplomatic Relations, entered into force in 1964, that states in Article 31 that “The immunity of a diplomatic agent from the jurisdiction of the receiving State does not exempt him from the jurisdiction of the sending State.” This points to the legal requirement for diplomats to respect regulations issued in their own state, which in some cases would include guidance on avoiding engagement with tobacco industry abroad. Article 41 of the Vienna Convention states that “it is the duty of all persons enjoying such privileges and immunities to respect the laws and regulations of the receiving State. They also have a duty not to interfere in the internal affairs of that State” (7). Considering that there are 182 Parties to the WHO FCTC, this flags the overall need of diplomats to respect the WHO FCTC obligations globally.

In addition to the recognized need for international collaboration to tackle the tobacco epidemic, countries which are Parties to the WHO FCTC commit to certain actions to fight the tobacco epidemic. The treaty particularly includes Article 5.3 that states that “In setting and implementing their public health policies with respect to tobacco control, Parties shall act to protect these policies from commercial and other vested interests of the tobacco industry in accordance with national law.” In Article 5.4, the treaty requires Parties to cooperate in formulating measures for its implementation. In Article 20.4.c it requires Parties to cooperate with international organizations to have a global system to collect and disseminate information on the tobacco industry (6).

In addition to the Articles included in the WHO FCTC that highlight the need for international collaboration and the necessity of this collaboration in countering the tobacco industry, since the treaty came into force, the Parties have decided to develop and progress its implementation in many Conferences of Parties, known as COPs. In the third COP, the implementation guidelines of Article 5.3 were adopted, which included that “Parties should not grant incentives, privileges or benefits to the tobacco industry to establish or run their businesses” and to interact with tobacco industry “when and to the extent strictly necessary to enable them to effectively regulate the tobacco industry and tobacco products” (8). The sixth COP decided to urge parties to “raise awareness and adopt measures to implement Article 5.3 and its implementing Guidelines among all parts of government including diplomatic missions” (9). Another decision was made in the sixth COP to require governments to make sure the tobacco industry doesn't influence international trade and investment instruments and to “take into account their public health objectives in their negotiation of trade and investment agreements” (10).

National regulations

Some countries have adopted national policies to regulate diplomats' interactions with the tobacco industry: the UK published guidelines for overseas posts on support to the tobacco industry (11); Australia issued guidance for public officials on interacting with the tobacco industry (12); and the United States has policies prohibiting actions such as promotion of the sale or export of tobacco or actions seeking the reduction or removal of non-discriminatory restrictions by foreign governments on tobacco product marketing (13).

Although monitoring diplomats' engagement with the tobacco industry is challenging, incidents of contact with the industry are documented by countries who are both Parties and Non-Parties to the WHO FCTC. High-level lobbying has been documented in Africa, Asia, Europe, and elsewhere, as tobacco companies have long received support from overseas missions (14–17). Although the incidents occurred in many countries, they have each been linked to one of the so-called “Big Four” transnational tobacco companies: Imperial Brands (previously Imperial Tobacco); British American Tobacco; Phillip Morris International; and Japan Tobacco International.

In this paper, we present a case related to BAT, one of the two transnationals based in the UK. It acts as a case study which helps to understand what diplomatic interactions with tobacco industry involve and might therefore guide further research looking at other instances of engagement by BAT and other transnational companies.

Early incidences of UK diplomats operating on behalf of tobacco companies have been identified in internal tobacco industry documents (18–23). These were before the WHO FCTC came into force. In recent years these continuing activities have been exposed *via* media investigations, questions in Parliament, and Freedom of Information (FOI) requests (1, 24–33). These show a strong public interest in examining how the diplomatic services engage with the tobacco industry. Examples of these activities by UK diplomats and others are summarized in **Box 2**. In this case study, an FOI request is used to investigate a case of a senior UK diplomat engaging with the tobacco industry.

Box 2 **incidents of diplomats engaging with tobacco industry.**

The United Kingdom

Incidents of British diplomats' direct interventions for the benefit of the tobacco industry, spanning decades from the 1960s to the 1990s, were recorded in Indonesia (20), Mexico, where the British Ambassador confirmed his “willingness to be orchestrated when requested” (21, 22), and Cambodia, where the British Ambassador was “keen to encourage a BAT investment” (23).

In 1991 the British ambassador in Argentina hosted a dinner for British business interests in the country with a chance to meet government officials. A Foreign Office minister was present and among those attending were representatives from BAT (34). A year earlier BAT reported that a lobbying effort to persuade the Argentinian government on easing tax rises was “supported by the British Ambassador” (18).

In 1992, when BAT wanted to buy a cigarette firm in Czechoslovakia, they received advice from the British Ambassador who, according to BAT, was “very well informed about the current situation.” However, it appeared that Phillip Morris International had already done the same: “[the British Ambassador] commented that the American Embassy were already claiming victory for Philip Morris.” Nonetheless the British Ambassador advised BAT on who they should lobby in the government (19).

Further incidents have been documented since the WHO FCTC came into force in 2005, and after the adoption of relevant COP decisions including the implementation guidelines of Article 5.3.

In 2012, the British Ambassador in Panama lobbied over tax increases and the impact of cigarette smuggling on BAT, “one of the most important British Companies” (27, 35). The tobacco industry often cites an increase in the illicit tobacco trade as an argument not to increase tax, an argument found to lack substance (36, 37).

There is a documented “global pattern of engagement” by British officials for the benefit of BAT (1, 2), a high profile example being in Bangladesh, a country with one of the highest prevalence rates of tobacco use. The British High Commissioner lobbied over several years on behalf of BAT in respect of a court case instigated in 2013 by the Bangladesh Board of Revenue over an alleged £170 million in unpaid sales tax (1, 24, 38, 39). The exposure of this activity by advocates in Bangladesh in 2017 led to UK media coverage and questions in Parliament (25, 26, 40, 41). However, the UK Minister of State for the Foreign and Commonwealth Office argued that the demands of the government of Bangladesh, was “discriminatory” against BAT (41, 42).

In 2015, diplomats were found to be supporting BAT's business interests in Pakistan, including attending meetings where BAT lobbied against plans for larger health warnings on cigarette packets (27–30, 43). Implementation of this policy was then delayed allowing BAT to comply, a success for another common industry tactic (44, 45). A more recent FOI confirmed that in 2020, staff from the UK high commission in Pakistan attended a promotional event for a BAT's new nicotine pouch product Velo (31).

In addition to interventions by diplomatic staff, FOI requests also showed that UK officials had repeated contact with tobacco companies in Panama, Venezuela, Laos, Cuba, and Burundi (26, 32, 33). However a government minister said: “The Government does not catalogue the representations it makes on behalf of companies” (42, 46). This appears contrary to requirements for transparency spelled out in the UK government's own guidelines. In addition, attendance at events organized or sponsored by tobacco companies is prohibited by the guidelines. Other fora, including meetings and events organized by local chambers of commerce and other business organizations, are attended by diplomats and mission staff. However, details are not usually publicly disclosed, and information provided in FOIs can be scant (26).

The United States

Over 30 years ago, in 1990, United States Trade Representative aggressively lobbied for American tobacco companies to access the Thai market (47). More recently, in 2017, the United States Ambassador to Vietnam endorsed the activities of the United States and the Association of Southeast Asian Nations Business Council. At the time, Phillip Morris International was vice-chair of its key committee on customs and trade, and led a trade delegation to meet with government officials, a delegation which included representatives of the tobacco company (48).

Switzerland

In 2019, the Swiss embassy officials lobbied the President of the Parliament of the Republic of Moldova, for Phillip Morris International to be given an opportunity to contribute to new tobacco legislation including on heated tobacco products in which the company has an interest (3, 49–51). Phillip Morris International donated funding for the inauguration party of the new Swiss Embassy building in Moscow the same year (52, 53).

Japan

Diplomats from Japan have helped tobacco companies to promote their activities in multiple countries. In 2015, the Japanese Ambassador to Ethiopia was present at the signing of the deal when Japan Tobacco International acquired 40% of the national tobacco company (54). Japanese missions have helped publicize tobacco company activities in Tanzania and Zambia (55, 56). In 2021, the Japanese Ambassador lobbied the government of Bangladesh on behalf of Japan Tobacco International criticizing taxation changes and other commercial factors impacting the tobacco company (57–59).

Germany

In May 2022, the German ambassador to Beirut, visited the offices of the “Regie,” the Lebanese Tobacco and Tobacco Inventory Administration, also present was the First Secretary for Financial Affairs who “was briefed on the achievements of the ‘Reggie’ in the fields of agriculture, industry, trade and others, and the societal role it has played in recent years” [translated from Arabic] (60).

## Methods

### Identifying the incident of this case study

Tobacco Tactics was launched in 2012 as an output from the Tobacco Control Research Group (TCRG) at the University of Bath. Since 2019, TCRG has been part of the global tobacco industry watchdog STOP. TCRG researchers regularly monitor and collect information on tobacco industry. The case studied in this paper was identified through media monitoring. Within a media article, there was a mention to a British ambassador attending the opening of an event celebrating an expansion of tobacco company in Jordan. There was not much information available in public domain about the incident or why the ambassador attended the event in contravention of national and international policies. Therefore, a decision was made to further investigate this incident.

### The methodology of investigation

Many countries have public access laws which give citizens the right to inspect documents held by their governing authorities. The exact nature of these access laws varies from country to another, but they generally allow any person to request a copy of any document held by government provided in doing so it doesn't breach confidentiality or other laws.

In the UK, the Freedom of Information Act was introduced in 2000 and came into effect in 2005. The Act excludes private companies, but information can be obtained in certain cases where they interact with the government. The presumption in the Freedom of Information Act is that material should be released but there are multiple exemptions that can be applied to justify withholding it. The Freedom of Information processes are overseen by the Information Commissioner's Office which can adjudicate on requests and ensure the Act is applied appropriately. Any person requesting information under the act has the right to appeal to the Information Commissioner's Office and if need be, to go all the way to the UK Supreme Court (the final court of appeal) if they believe the law has been wrongly applied (61). In this paper, an FOI request was used as the main tool to further investigate this incident of a British ambassador attending an event celebrating the expansion of a tobacco company in Jordan. The process included requesting an internal review by the Foreign, Commonwealth and Development Office, and a formal complaint to the Information Commissioner's Office, the UK's independent authority set up to uphold information rights in the public interest.

## Results: UK ambassador engaging with tobacco industry in Jordan and Yemen

The UK signed the WHO FCTC Treaty in 2003 and ratified it in 2004. In 2016, the UK initiated the FCTC 2030 project to support LMICs in tobacco control and implementation of the WHO FCTC (62). There are strong arguments that the UK diplomats' engagement with the tobacco industry is incompatible with the UK's leadership in global tobacco control. In 2019, the UK was placed number one in the Global Tobacco Industry Interference Index, a report which ranks countries worldwide based on how well they implement and comply with Article 5.3 and its implementation guidelines (63).

However, the UK has failed to maintain such a position in the second and third editions of the Index (64). Diplomats appear to have repeatedly lobbied with the tobacco industry in LMICs in these instances (1). The incident related in this case study is not only evidence such behavior is ongoing but it also indicates a concerning lack of transparency when it comes to monitoring the UK's commitment to international treaties.

TCRG researchers found a news article (65) about the Kamaran company in Yemen (66), which is 31% owned by BAT and partially Yemen state-owned (67). The article presented a document that shows Kamaran gave money to a newspaper for the role played by the newspaper in combatting the illicit tobacco trade, and for supporting national companies, after the newspaper asked for money. The article also revealed how the company engaged in the civil war in Yemen and funded an armed group. In addition, it mentioned that the British ambassador attended the opening of an event celebrating an expansion of the company in Jordan.

A Kamaran press release found on its website, in Arabic, celebrated the opening of a new factory in a free trade zone in Jordan. This allows the factory to benefit from tax and customs relief. The company reported that officials from three countries attended the opening, including the British ambassador to Yemen, the Jordanian ambassador to Yemen and the Yemeni ambassador to Jordan (68). Further investigation identified a video (69) that shows (at 9:45) that Michael Aron (70), the then British Ambassador, attended the opening of the BAT's factory and was interviewed by the media. He highlighted in the video that BAT has investments in Yemen and this investment would benefit both BAT and Yemen. It is worth noting that this factory is in a free trade zone in Jordan, within the airport area, that provides multiple incentives to investors, as detailed in the statement of a Jordanian official in the same video.

As part of the investigation of this incident, the TCRG submitted an FOI request in September 2021 to the British Foreign, Commonwealth and Development Office requesting a list of all contacts UK embassy officials had had with tobacco companies in Yemen and Jordan for the period from 1 January 2018 to 29 September 2021, including incidents of diplomats attending or arranging meetings or functions and responding to correspondence or phone calls from the tobacco industry.

An initial response was received from the Foreign, Commonwealth and Development Office stating that the information would be provided by October 2021, then repeated communications were received delaying the response monthly for 4 months. The TCRG responded stating that it was minded to refer the whole issue to the Information Commissioner's Office. A response was finally received in February 2022, 5 months after submitting the initial FOI. However, the response failed to mention the incident when the UK ambassador engaged with the tobacco industry, and the Section 43 exemption on commercial confidentiality was used. The response also discloses that diplomats invited BAT to a British-Jordanian government event “the London Initiative,” an international conference on trade and investment to be held in London in February 2019 (71–73). This invitation does not align with the WHO FCTC, as it is not an interaction necessary for the regulation of the tobacco industry and its products.

Within the same month, February 2022, an internal review was requested by the TCRG arguing that contacts between government officials and the tobacco industry are governed by the WHO FCTC to which the UK is a Party, and that the treaty requires Parties to limit contacts with the tobacco industry, to be transparent about any contacts and to report and disclose any contacts. The TCRG clarified that these discussions with the industry are of public interest not just in the UK, but also in the Middle East Region which has its own obligations to the WHO FCTC. The TCRG pointed out that the UK is not demonstrating leadership or good practice in the region by withholding information on contacts with the tobacco industry. The TCRG also highlighted that the FOI request asked for contacts between embassy officials in Yemen and Jordan and the tobacco industry between 1 January 2018 and 29 September 2021, however the response ignored the incident of the British Ambassador Michael Aron attending the opening of a new factory for the Kamaran company within the requested period.

A response was received from the Foreign, Commonwealth and Development Office in April 2022 stating that “… I am therefore satisfied that this exemption is engaged. The public interest arguments for and against release were also set out in detail in the response and I find that the balance of the public interest lies in favor of maintaining the exemption.” A complaint to the Information Commissioner's Office was submitted within same month by the TCRG, and a response in May 2022 from the Information Commissioner's Office stated that the case was eligible for investigation and that a case officer would be allocated.

In December 2022, the Information Commissioner's Office decided that the Foreign, Commonwealth and Development Office should “disclose the information previously withheld under Section 43.” It also decided that in failing to respond and disclose all non-exempt information within 20 working days of receipt of the request, the Foreign, Commonwealth and Development Office breached Section 10 (time for compliance with request) of the Freedom of Information Act.

The decision referred to the Foreign, Commonwealth and Development Office stating that the ambassador attended the event, opening the factory, but that “no formal record of the event was recorded and that there was no briefing prepared ahead of the event,” and that the Foreign, Commonwealth and Development Office “would only record details of more formal meetings and not receptions/launch events such as this.” The decision number is IC-167611-D5S9 and is available at https://ico.org.uk/.

In December 2022, after the Information Commissioner's Office decision, the Foreign, Commonwealth and Development Office shared information which it had previously withheld: “We were approached by East of England Trading Co (UK company owned by a British Jordanian) to support them in a commercial conflict they had with the Government of Jordan related to their tobacco shipment that was held at their warehouses at Aqaba Free Zone. FCDO were unable to offer support as it was tobacco related.”

## Discussion

In this case study, it is challenging to identify the extent of contravention of national and international policies, given the lack of information and transparency around government engagement. However, with the limited available data, the following points can be highlighted.

- The engagement between government officials with the tobacco industry appears to be a violation to the implementation guidelines of Article 5.3 of the WHO FCTC principle that limits interactions only to the extent necessary to regulate the tobacco industry and its products. Attending a factory opening does not seem to meet this standard of a necessary meeting.
- The fact that the tobacco manufacturer is established in a free trade zone with incentive provided to investors raises questions on the compliance to the implementation guidelines of Article 5.3 of the WHO FCTC principle requiring no preferential treatment or incentives to be given to the tobacco industry.
- The lack of information about the support provided by the UK ambassador to the tobacco industry and the initial use of the Section 43 exemption, despite a public interest consideration in how it should be applied; the final confirmation by the Foreign, Commonwealth and Development Office that the ambassador attended the event; and the statement that such incidents are not regularly reported all contradict the implementation guidelines of Article 5.3 of the WHO FCTC principle that requires the Parties to be transparent when interacting with the tobacco industry. Similarly:
- The Foreign, Commonwealth and Development Office response used the Section 43 exemption that applies in trade situations, even though the COP to the WHO FCTC decided before in decision FCTC/COP6 (19) that Parties are required to make sure the tobacco industry doesn't influence international trade and investment instruments and governments “take into account their public health objectives in their negotiation of trade and investment agreements.”
- The fact that this manufacture engages Yemen, a country in emergency, contradicts the WHO FCTC COP decision FCTC/COP8 (20) that requires Parties facing complex emergencies to continue to fulfill their obligations under the WHO FCTC to the extent possible, and more importantly…. to pay special attention to Article 5.3 of the WHO FCTC and related Guidelines.
- The participation of the UK ambassador in the factory opening appears to contradict the UK's revised guidelines for overseas posts on support to the tobacco industry. The guidelines clearly state that “Posts must not:... Attend or otherwise support receptions or high-profile events, especially those where a tobacco company is the sole or main sponsor and/or which are overtly to promote tobacco products or the tobacco industry (such as the official opening of a UK tobacco factory overseas).”
- In addition to the violations above related to the incident of opening a tobacco factory, the Foreign, Commonwealth and Development Office response to the FOI disclosed that diplomats invited BAT to a British-Jordanian government event “London Initiative,” another case which appears to contradict requirements of Article 5.3 of the WHO FCTC.

The Foreign, Commonwealth and Development Office final response stated that when asked for support by a tobacco company, they “were unable to offer support as it was tobacco related.” This implies that they are aware of the specific requirements when it comes to dealing with the tobacco industry. This situation raises two fundamental questions: did the ambassador attend the opening despite knowing this is against the national guidance and the international treaty guidelines? And if so, what power or influence does the tobacco industry have to make a UK ambassador act in contrary to his government's guidance and the obligations of an international treaty?

For countries like the UK that want to play a leading global role in tobacco control, the whole government should be aligned to this purpose. In this case study, it seems that while the UK funded tobacco control activities in Jordan through the FCTC 2030 project, its diplomats were engaging with a tobacco company which was expanding in the country at same time. This contradiction raises a question around the UK's priorities specifically in Yemen and Jordan, and in LMICs more generally. Does the UK want to support BAT or promote public health? The UK's image as a global public health leader is further tarnished when the British ambassador engages with a tobacco company accused of fuelling civil war. The sensitivity about such actions is especially acute in a region where the UK has a long colonial history. Making economic threats, as in the case of the Japanese diplomats in Bangladesh, indicates that some governments might use their diplomats to intimidate those acting against big tobacco's corporate interests.

The tobacco epidemic was recognized as a global threat to public health decades ago. The need for international collaboration in tackling tobacco use was identified as a necessity in the WHO FCTC treaty almost two decades ago. Diplomats' behavior in engaging the tobacco industry is a major risk for such global collaboration and undermines global public health efforts to address the epidemic. There is a huge concern around equity when a country does very well in tobacco control within its own borders, but still supports the tobacco industry overseas. Prioritizing one country's own economic benefit over another's public health is just not fair.

Discriminatory actions against the tobacco industry are a right given by international law to governments as a tool to be used in tackling the tobacco epidemic. For example, the implementation guidelines of Article 5.3 of the WHO FCTC urge Parties to exclude the tobacco industry from any health setting related to tobacco control and it urges Parties to decline corporate social responsibility activities by the tobacco industry. The treaty does not consider the tobacco industry as a normal industry, but describes it as the vector of the tobacco epidemic. Therefore, diplomats' actions in supporting the tobacco industry helps to potentially normalize the tobacco industry a business like any other, contrary to public health measures and aims.

The fact that the information about diplomats' engagement with the tobacco industry is very challenging to get, and that such interactions are sometimes protected by legal exemptions from disclosure, creates an extra burden on Parties and civil society in their efforts to fulfill the WHO FCTC treaty requirements of monitoring and exposing the tobacco industry. Investigating the case study presented in this paper took from August 2021 to December 2022 to be concluded.

The British ambassador claimed in media that there are benefits to Yemen from tobacco industry expansion and from BAT's presence. This claim coming from a high official is an action which undermines public health efforts in the host country. While the WHO FCTC treaty bans corporate social responsibility to avoid giving the industry the image of being a responsible partner, such behavior from the diplomat contradicts the core aim of the treaty and undermines its value.

## Conclusion and recommendations

We have set out in this paper a case study of the activities of diplomats which strongly suggest that the both the spirit and the rules of the WHO FCTC have been breached. We have also set out a brief historical context to show that there is a much longer history in many countries of the diplomatic corps serving tobacco industry needs. It is fair to say that the business of governments abroad is very often business—representing the interests of domestic corporations which employ voters, pay taxes and make donations, and can wield political influence. Nonetheless, other commercial activities are not regulated by the WHO FCTC and the treaty is clear on what is and what is not prohibited in this area. Our case study, we suggest, is not technical transgression in isolation but a pattern of egregious subversion of an international treaty.

We call the Parties to the WHO FCTC to confirm the extent of Article 5.3 to ensure that government representatives abroad abide by its provisions as closely as they would at home. The COP guidance could give particular focus to this issue and require that both the home and the hosting country disclose any information related to tobacco industry discussions to the public through its relevant transparency processes. Therefore, if a diplomat wanted to send a lobbying letter to a minister in their host country, they should expect that this letter to be publicly available. By acting in this way, the guidance is not identifying particular countries or particular companies—it is merely ensuring that the same effective level of activity is demanded globally.

At a national level, we call the governments to raise awareness on policies and procedures to be followed abroad when dealing with the tobacco industry. We strongly recommend adoption of standardized procedures to report on any interaction between diplomats and tobacco industry. A template that includes specific details—who, what, why, where, how, and the outcome of any interaction—should be completed for all interactions and disclosed.

The information gathered here was available from public sources using different methods but none of which required special status. The use of public access laws to extract information and hold governments and the industry to account is one which we feel could be deployed more effectively. Tobacco control advocates might wish to consider how they could use this research, what support they might need from the global community and how this information could be shared to have maximum impact. There is a real opportunity here to intensify the spotlight on tobacco industry interference in this particular sphere.

## Author contributions

RA identified this case study, conducted the investigation including the processes of FOI, internal review, and the Information Commissioner's Office investigation requests and prepared the manuscript. KS contributed to the writing and reviewing of the manuscript providing an overview of the similar incidents globally. PC provided guidance to the investigation process and contributed to the writing and reviewing of the manuscript. All authors read and agreed on the final manuscript.
